# ME-Work: Development and Validation of a Modular Meaning in Work Inventory

**DOI:** 10.3389/fpsyg.2020.599913

**Published:** 2020-12-07

**Authors:** Tatjana Schnell, Carmen Hoffmann

**Affiliations:** ^1^Existential Psychology Lab, Institute of Psychology, University of Innsbruck, Innsbruck, Austria; ^2^Psychology of Religion and Existential Psychology, MF Norwegian School of Theology, Religion and Society, Oslo, Norway

**Keywords:** meaningful work, meaningless work, source of meaning, coherence, belonging, significance, purpose, burnout

## Abstract

As research on meaning in work progresses, access to theoretically integrated, differentiated survey instruments becomes crucial. In response to this demand, the present article introduces ME-Work, a modular inventory to measure meaning in work. Derived from research findings on meaning in life, the ME-Work inventory offers three modules that can be used separately or jointly. Module 1 assesses four facets of meaning in work, i.e., coherence, significance, purpose and belonging; module 2 measures the subjective assessment of work as meaningful or meaningless, and module 3 records the extent to which work is perceived as a source of meaning. We report on the development of the instrument and the results of an exploratory factor analysis in a pilot study of 115 working adults. A further study with 278 working adults provided evidence for construct and incremental validity. Relationships with meaning in life, mental health, job satisfaction, socio-moral climate, burnout and work as meaning were investigated. Confirmatory factor analysis supported the factor structure. Gender-specific analyses of the four facets of meaning’s differential predictive power provided additional insights. Practical implications and further research needs are discussed.

## Introduction

Among ancient Greeks and Romans, gainful employment was not well regarded. Above all, it was trouble and a burden. It was even considered as morally reprehensible when not motivated by the product or service itself but by the acquisition of money (Aßländer, 2005). Nowadays, moral reprehension is experienced by those who do not work (Krug et al., 2019). Our profession is at the core of our identity. In a United States Gallup poll, 55% of workers said they got a sense of identity from their job; among college graduates, this was the case for 70% (Riffkin, 2014). However, there seems to be a change in awareness of what work means to us. While earning a living is still the basis of most occupations, a profession is no longer seen as a mere necessity. Instead, the focus is more and more on the fulfilling aspects of work, often associated with calling (Wrzesniewski et al., 1997), or purpose and meaning (Yeoman et al., 2019; Savvides and Stavrou, 2020). The present article focuses on the latter: meaning in work, and how it can be differentiated and measured. We introduce a modular inventory that measures the extent to which work is perceived as meaningful or meaningless, or even as a source of meaning in life. If, in addition, a more in-depth analysis of possible reasons for experiencing meaning or its lack is desired, a further module is available. It assesses the subjective perception of four central facets of meaning at the workplace: significance, purpose, coherence and belonging.

### Why and When Meaningful Work Makes Sense

Over 40 years ago, researchers started investigating beliefs, expectations, attitudes and experiences related to work, or unemployment, especially in industrialized societies. Hackman and Oldham (1975) proposed meaningfulness as one of several psychological states that mediate job design features and work outcomes. Jahoda (1982) counted a sense of *collective purpose* as one of five latent functions of employment that contribute to employees’ mental health, but create a sense of purposelessness when work is lacking. On an international level, the MOW (Meaning of Work) research program conducted large-scale studies to explore how people’s lives were mentally affected by paid and self-employment (Ruiz-Quintanilla and Claes, 2000). Since then, research on meaning in, of and at work has contributed to an ever-growing body of insights. Bailey and Madden (2020) give an overview of the history of meaningful work in psychology, philosophy, ethics, sociology and management studies; Lysova et al. (2019) provide a multi-level review and integration of findings with regard to fostering meaningful work in organizations. Although interpretations of meaningful work still vary, data suggest that work, when perceived as meaningful, has positive consequences for employees and employers alike. A large number of studies prove such positive connections (for a meta-analysis, see Allan et al., 2019). Meaningful work is related to a general sense of meaning in life (Steger and Dik, 2009; Steger et al., 2012; Duffy and Dik, 2013; Schnell, 2018), work engagement (Höge and Schnell, 2012), turnover intentions (Arnoux-Nicolas et al., 2016), depression (Steger et al., 2012; Daniel, 2015), socio-moral organizational climate (Schnell et al., 2013; Höge and Weber, 2018; Schnell, 2018), job satisfaction (Duffy and Dik, 2013; Duffy et al., 2013, 2014; Allan et al., 2018; Schnell, 2018; Bailey et al., 2019; Rothausen and Henderson, 2019), and health experience (Lease et al., 2019), although for the latter there are indications of gender-specific differences (Dich et al., 2019).

To sum up the scientific evidence, it can be said that people who see an intrinsic value in their work and enjoy doing it also tend to perform their work conscientiously, responsibly and with quality. This is undermined when organizations themselves have lost sight of the purpose of their products or services; when neoliberal economic principles have moved so much into the foreground that criteria such as meaningfulness, quality, sustainability and social responsibility fall behind (Crowley and Hodson, 2014). It is similarly detrimental to meaningful work when organizations instrumentalize strategies to increase meaning in work for performative intent (Bailey et al., 2017). The meaning of work, then, “is reduced to a mere transaction between two parties, thereby neglecting the intrinsic meaning of work and employment relationships for people” (Bal and Dóci, 2018, p. 538). As a consequence, many employees suffer from disillusionment. Above all, people who started their careers with idealism and enthusiasm are affected. “What started out as important, meaningful, and challenging work becomes unpleasant, unfulfilling, and meaningless. Energy turns into exhaustion, involvement turns into cynicism, and efficacy turns into ineffectiveness” (Maslach et al., 2001).

### A Widespread Quest for Meaningful Work

In a 2019 survey, Xing asked over 22,000 employees whether they would be willing to switch to a new job with more meaning or social responsibility if it meant earning less money: 50% of Germans, 49% of Austrians and 62% of Swiss participants responded “yes” (XING, 2019). The Nuremberg Institute for Market Decisions and St. Gallen Symposium surveyed more than 1,000 future top talents (international young scientists, entrepreneurs and politicians aged around 30) about the importance of the question of meaning for their choice of career. Twenty-six percent reported having rejected a job offer because of a conflict of values; 40% had opted against applying to an interesting employer because of a conflict of values; 42% had decided to accept a lower salary by choosing a more meaningful job, and 63% said they were searching for meaning-oriented employers (Nuremberg Institute for Market Decisions and St. Gallen Symposium, 2019). Many other current national and international surveys confirm these figures (cf. Schnell, 2020). But what is it exactly people are looking for when they search for meaning in their work lives?

### Facets of Meaning in Life Inform Facets of Meaning in Work

When asked to rate the meaningfulness of their job, people can usually respond without further instructions. But what are the underlying criteria for this assessment? In a recent review, Bailey et al. (2019) come to the conclusion that meaningfulness at work is a complex and multidimensional construct; “however, uncertainty remains over which dimensions of meaningfulness should be included and which are most salient” (Bailey et al., 2019, p. 99). A reference to research on meaning in life can be instructive here, where four central features of meaning have emerged over the last few years. These are significance, purpose, coherence and belonging (Schnell, 2014, 2020). *Significance* means the perceived impact of personal action, or non-action. *Purpose* refers to the availability of a direction, serving as a compass when it comes to making decisions and choosing goals. *Coherence* describes a sense of comprehensibility and consistency. *Belonging* means perceiving oneself as part of something larger than the self, as having a place in this world (Schnell, 2020). Concerning the inclusion of belonging as a central feature of meaning in life, disagreements still exist at present. While some authors negate its significance for the construct of meaning (George and Park, 2016; Martela and Steger, 2016), there is ample evidence for an inherent connection (Stillman and Baumeister, 2009; Lambert et al., 2013; Lund et al., 2019), which often becomes apparent precisely when belonging is missing (Lyons-Padilla et al., 2015; Saarelainen, 2018). The concept of social alienation, describing a sense of disconnection from society, other people, or one-self, also highlights this link between not-belonging and meaning by defining “meaninglessness” as one of the core features of social alienation (Seeman, 1959; Jaeggi, 2014).

In the context of research on meaningful work, the importance of belonging does not seem to be questioned. Independent operationalizations of all four facets – significance, purpose, coherence and belonging – have been shown to empirically predict meaningful work (Schnell et al., 2013). They also overlap with Rosso et al.’s (2010) influential theoretical framework describing four major pathways to meaningful work, individuation, contribution, self-connection and unification. *Individuation* is associated with experiences of the meaningfulness of one’s action, hence self-efficacy (Rosso et al., 2010). This definition is closely related to the above described concept of significance as perceived impact of personal action. In the study by Schnell et al. (2013), however, self-efficacy did not contribute significantly in the final model, whereas task significance – the perceived impact of one’s work – did. Rosso et al.’s (2010) *contribution* is paraphrased with purpose and transcendence and thus explicitly linked to the above suggested concept of purpose. *Self-connection* in Rosso et al. (2010) is explained as denoting authenticity, e.g., with regard to self-concordance and identity affirmation. This aspect is well covered by our concept of coherence. Finally, *unification*, in Rosso et al.’s (2010) terms, is realized through belongingness, which again links directly to the fourth facet of meaning, belonging.

In the context of meaningful work, *significance* refers to an awareness that one’s work positively benefits other people or society as a whole (Grant, 2008; Allan, 2017). *Purpose* at the workplace can range from being primarily oriented by shareholder expectations to contributing to a higher cause which transcends the organization. Meaningful work is typically associated with contributing to a higher, or self-transcendent, purpose at work, be it secular (Schnell et al., 2013) or spiritual (Lips-Wiersma, 2002). This can either be inherent in the kind of work, such as that by many non-profit organizations (Dempsey and Sanders, 2010), or in additional responsibility taken by an organization, such as corporate social responsibility (CSR; Crane et al., 2019). Perceived CSR has been linked to employees’ sense of meaning at work (Glavas and Kelley, 2014), especially for younger employees (Supanti and Butcher, 2019). *Coherence* refers to an alignment between work requirements and a person’s characteristics, skills and scheme of life. Such work-role fit has repeatedly been shown to be relevant for meaningful work (May et al., 2004; Bunderson and Thompson, 2009; Schnell et al., 2013). Finally, *belonging* represents one of the core mechanisms of meaning in work in the influential review by Rosso et al. (2010) and – as “unity with others” – one of four quadrants in Lips-Wiersma’s Map of Meaning (2017). It arises from being part of a group or team at work, where acceptance, acknowledgment and recognition arise and allegiance is strong (McClure and Brown, 2008; Schnell et al., 2019). Numerous studies have reported links between varieties of operationalizations of belonging and meaningful work (e.g., Liden et al., 2000; Lips-Wiersma and Morris, 2009; Pavlish and Hunt, 2012; Schnell et al., 2013; Geldenhuys et al., 2014; Bailey and Madden, 2016; Höge and Weber, 2018).

The proposed four facets of meaningfulness in work provide fine-grained insights into the experience of meaning. Another important perspective in research on meaning in life focuses on the sources people draw on to find or construe meaning in their lives (Schnell, 2009, 2011). Sources of meaning are defined as purposes or orientations that give meaning to life when being actively pursued; they are meaning in action (Leontiev, 1982; Schnell, 2004/2009). While in theory nearly all occupations can be perceived as meaningful, this does not apply when it comes to perceptions of work as a source of meaning. The focus here is not on the work experience itself, but on what an occupation can contribute to a person’s life as a whole. If we succeed in bringing our personal meaning to bear in work, if we can design working tasks and conditions in accordance with our personal values and thus self-realize and grow in and through work, then it can serve as a source of meaning. It is probably here that we find the closest connection between meaningful work and the construct of calling: “People with Callings find that their work is inseparable from their life. A person with a Calling works not for financial gain or Career advancement, but instead for the fulfilment that doing the work brings to the individual” (Wrzesniewski et al., 1997, p. 22). However, it also represents a double-edged sword, since it has repeatedly been associated with personal self-exploitation or exploitation by employers (Bunderson and Thompson, 2009; Dempsey and Sanders, 2010; Cardador and Caza, 2012; Bailey and Madden, 2017; Schnell, 2018; Schnell et al., 2019).

### Measuring Meaning in Work

When it comes to instruments to measure meaningful work, theoretically embedded differentiations are still rare (Bailey et al., 2019). According to a recent review of scales that measure meaningful work (Both-Nwabuwe et al., 2017), only two instruments offered a multidimensional approach and were sufficiently validated: the Comprehensive Meaningful Work Scale (Lips-Wiersma and Wright, 2012) and the Work and Meaning Inventory (Steger et al., 2012).

Lips-Wiersma and Wright’s Comprehensive Meaningful Work Scale (2012) was developed on the basis of two qualitative studies. Its process-oriented scales measure four dimensions of meaningful work, the dynamic tensions between these, inspiration and reality. The four dimensions and three additional scales allow for a complex and differentiated assessment of the dynamic process of finding meaning in work. Two of the above proposed facets of meaning – purpose and belonging – are present in the CMWS, operationalized as serving others and unity with others. A concept of coherence is also included, operationalized as seeking a balance between self and others and between being and doing, but not referring to the broader fit between a person’s job and their characteristics, skills and general life scheme. The 28-item instrument does not provide a general assessment of perceived meaning at the workplace, or of its lack. Neither does it allow insight into whether a person experiences their job as a source of meaning.

The Work and Meaning Inventory by Steger et al. (2012) is a short (10 items) and widely used instrument (Both-Nwabuwe et al., 2017). It contains a general measure of meaningful work (positive meaning), plus two other scales called greater good motivation and contribution to meaning-making. The authors do not explain why they selected exactly these three dimensions and how this choice might be linked to a theory of meaningful work. The proposed three-factor structure could not be replicated in the German translation of the scale (Harzer and Steger, 2012). Data from other translations also deviated from the suggested factor structure (Puchalska-Kamińska et al., 2019).

Moreover, as noted by Bailey et al. (2019), the focus of all measures so far has entirely been on meaningful work as a subjective experience. Experiences of meaning, we would like to add, cannot be negotiated exclusively within the individual. Perceiving meaning is perceiving more than what is – i.e., seeing a surplus of a thing, an act, a situation (Schnell, 2020). Other than a feeling of enjoyment, e.g., an assessment of meaning involves the consideration of a higher level providing the “why,” the reason, or the goal of the thing, act, or situation. (For example: Producing X is meaningful because X is useful to Y; task X is meaningful because it results in Y; doing X with my colleagues is meaningful because it adds up to a joint creation of Y; working for company X is meaningful because the company takes responsibility for Y, etc.). It is therefore informative to include further levels of reference to social, institutional and societal contexts in an assessment.

## ME-Work: A Modular Inventory to Measure Meaning in Work

The modular inventory of meaning in work (ME-Work) presented here strives to take these desiderata into account and responds to an “urgent[ly] call for using a comprehensive definition of meaningful work and corresponding validated meaningful work scales in empirical studies in paid work contexts” (Both-Nwabuwe et al., 2017, p. 13). In structure and content, the ME-Work is based on a theory of meaning in life (Schnell, 2014, 2020), thus providing a connection of the more specific field of meaning in work to insights from meaning in life research. It further covers various levels of evaluation: the subjective sense of meaningfulness of one’s work (meaningful work), the perceived meaninglessness of one’s work (meaningless work), the potential function of work as contributing to meaning in life (work as a source of meaning) and the underlying facets, or mechanisms, of meaning (significance, coherence, purpose and belonging). Meaningful and meaningless work assess a person’s perception of the presence or lack of meaning in their work; work as a source of meaning refers to a person’s perception of their work as contributing to their fulfilment, personal growth and self-actualization. The four facets of meaning reach beyond the subject. Significance measures an awareness of the positive effects one’s work has for others, or society as a whole. Coherence focuses on the assignment and organization of tasks in relation to a person’s character, interests and general life design. Belonging refers to exhibiting and perceiving allegiance and acknowledgment at the workplace. Purpose introduces an external criterion to the concept, by operationalizing the perception of one’s employer as socially responsible. Due to the inventory’s modular character, the scales can be used in whole or in part as required, which is beneficial to the claim to economy.

The ME-work can thus contribute to a series of issues identified as relevant (Bailey et al., 2019), such as the relative significance of the various facets of meaningfulness to positive and negative experiences at the workplace and beyond. The present article refers specifically to meaning in life, job satisfaction, professional efficacy and socio-moral climate as positive experiences, and general mental distress, emotional exhaustion and cynicism as negative experiences. These questions will be examined after presenting the development of the inventory (Study 1) and evidence for its construct, factorial and incremental validity (Study 2).

### The Development of ME-Work

Building on its predecessor, the Meaningful Work Scale by Schnell and Höge (Höge and Schnell, 2012; Schnell et al., 2013; Littman-Ovadia and Lavy, 2016; Glaser et al., 2017; Littman-Ovadia et al., 2017; Pollet and Schnell, 2017; Höge and Weber, 2018; Hulshof et al., 2020), the ME-Work inventory is intended to capture the facets of significance, coherence, purpose and belonging as separate scales, in addition to general assessments of one’s own work as meaningful, meaningless and source of meaning. These distinctions are the basis for three modules that can be used individually or jointly: (1) facets of meaning in work, (2) meaningful work and meaningless work, and (3) work as a source of meaning. Two versions – for employees and for self-employed persons – were developed.

Ten researchers who were familiar with the constructs and related research generated items for the above constructs. Seven of them rated the ensuing 107 items with regard to their comprehensibility and construct centrality. Both item generation and item ratings were carried out independently. ICC estimates and their 95% confident intervals were calculated based on a mean-rating (*k* = 7), absolute-agreement, 2-way mixed-effects model. Absolute agreement was fair, with 0.59 (CI 95% = 0.47–0.70) for comprehensibility and 0.52 (CI 95% = 0.37–0.65) for centrality (Koo and Li, 2016). The mean comprehensibility rating (response format 0–5) was 4.21 (SD = 0.57), the mean rating for centrality was 4.08 (SD = 0.57). Items for inclusion in the pilot study were selected on the basis of the summed up comprehensibility and centrality scores on the one hand, and further theoretical discussion on the other hand. This referred to differences in the evaluation and aimed at ensuring content breadth through different perspectives and not too similar wording. For the scales purpose and belonging, items for freelancers were formulated analogously to the items for employees. The items for meaningful work and meaningless work were taken from the already existing Meaningful Work Scale (Höge and Schnell, 2012; Schnell et al., 2013).

## Pilot Study

### Procedure

We constructed an online survey protocol comprising the ME-Work items and demographics. Using the online survey software SoSciSurvey, a broad range of working people were invited to participate via newsletters, internet forums and social media platforms. We targeted both employed and self-employed workers. Completion of the survey took between five and ten minutes. Participants received no incentives. Ethical approval for the pilot and validation study was issued by the Review Board (Psychology) of the University of Innsbruck.

### Participants

A total of *N* = 149 participants answered the survey, of whom *n* = 115 were employed and *n* = 14 were self-employed. For further statistical procedures, only the (*N* = 115) employees were analyzed due to the small number of self-employed participants. Therefore, only the questionnaire version for salaried employees was examined in more detail. The mean age was 34, ranging from 20 to 68 years. Fifty-four percent of the sample were female. The sample leaned toward higher education, as 85% had a high school diploma or higher qualification. Of *N* = 115 participants, *n* = 60 worked full-time, *n* = 35 part-time, *n* = 16 participants worked in marginal and *n* = 4 in another form of employment. A large proportion of the participants (30%) worked in the educational and social sector, 14% in the health sector, 13% in business and administration, 5% each in the hotel and restaurant industry, in sales and in transport and logistics, 4% each in nature-related professions, in construction, in metal and machinery, in IT and in culture. The remainder came from the sectors of design and art, vehicles, planning and construction, food, printing and building services engineering.

### Measures

An online questionnaire was used to assess demographic variables and the initial version of ME-Work consisting of 39 items (25 items to measure the four facets of meaning and 14 items to measure meaningful work, meaningless work and work as a source of meaning). ME-Work items were answered on a six-point Likert scale from 0 (strongly disagree) to 5 (strongly agree). The following demographic and work-related variables were assessed: age, gender, education, professional activity (yes/no), type of work (self-employed or employed), average weekly working hours and professional sector.

## Results

### Internal Consistencies

Reliability analyses showed high internal consistencies according to Cronbach’s Alpha. All items had item-total correlations >0.40 (Nunnally, 1967). Wherever it was possible to increase the economy of scales without compromising reliability, we reduced the number of items per scale to three. Like that, 22 items remained. The following scales with three items each resulted: coherence (α = 0.91), significance (α = 0.92), belonging (α = 0.88), meaningful work (α = 0.88), meaningless work (α = 0.78) and work as a source of meaning (α = 0.84). The purpose scale (α = 0.80) kept four items, as a reduction to three items would have been accompanied by a significant decrease in reliability. Overall, internal consistency can be rated as good to very good.

### Exploratory Factor Analysis

To clarify the structure of the ME-Work, two exploratory factor analyses were conducted. Principal axis factoring with oblique rotation (Tabachnick et al., 2007) was employed. The goal of the first principal factor analysis (PFA) was to highlight the structural aspects of the conceptual mechanisms of meaning. Thirteen items were included. The Kaiser-Meyer-Olkin (KMO) test yielded a coefficient of 0.88, indicating that the data were very well suited for factor analysis. Communalities were high (7 > 0.70, 3 > 0.60, 1 > 0.50, 2 < 0.50), so that the requirements for conducting exploratory factor analysis with a sample size of *N* = 115 were met (cf. Bühner, 2011). In line with the theoretical assumptions, we found a four-factor structure that represented the four facets of meaning in work, i.e., coherence, significance, purpose and belonging. Whereas the Kaiser criterion suggested a three-factor solution (eigenvalues 6.30, 2.02, 1.18, 0.78), the extraction of four factors resulted in a clear structure with all items loading in accordance with their theoretical conception. Loadings on the respective factors were strong and displayed no cross loadings >0.30. Factor loadings for coherence ranged from *r* = 0.60 to 0.86, for belonging from *r* = 0.68 to 0.90, for purpose from *r* = 0.53 to 0.79 and for significance from *r* = 0.77 to 0.83.

The factor correlation matrix contained correlations between factors >0.32, thus indicating that an oblique rotation was appropriate (Tabachnick et al., 2007). In total, the four factors explained 79% of the variance. The rotation sums of squared loadings represented an even distribution over the four factors (coherence = 4.13, belonging = 3.73, purpose = 4.00, significance = 4.13). The PFA thus confirmed the four-dimensional solution as theorized (Höge and Schnell, 2012).

The second PFA tested the factor structure of the other two modules of ME-Work: meaningful work, meaningless work (module 2) and work as a source of meaning (module 3). The KMO coefficient was 0.91, confirming that the data were excellently suited for factor analysis. Communalities suggested feasibility of factor analysis (4 > 0.70, 3 > 0.60, 1 > 0.50, 1 < 0.50). The Kaiser criterion suggested a one-factor solution (eigenvalues 5.72, 0.85, 0.70). To better represent the theoretical conception, we decided to extract two factors. Together, they explained 67% of the total variance. Rotation sums of squared loadings were 4.93 and 4.56. The first factor represented work as a source of meaning, with factor loadings ranging from *r* = 0.64 to 0.97. The second factor represented the second module of the ME-Work, meaningful and meaningless work, with factor loadings ranging from *r* = 0.35 to 0.96. An examination of item loadings revealed that one item assumed to measure meaningful work loaded strongly on the factor representing work as a source of meaning; it was thus assigned to this scale. Another item from the meaningful work scale showed a rather weak loading. We therefore generated two additional items to measure meaningful work. The enhanced scale’s fit was evaluated in the validation study (see below). This second principal axis factor analysis legitimized the conceptual separation of the two modules, meaningful/meaningless work and work as a source of meaning.

### Validation Study

To validate the ME-Work, we conducted a large-scale study among working people.

#### Procedure

We constructed an online survey protocol comprising the ME-Work items as described above, together with demographics and several scales to examine the inventory’s construct validity. Using the online survey software SoSciSurvey, a broad range of working people were invited to participate via newsletters, internet forums and social media platforms. Completion of the survey took between 10 and 15 min. Participants received no incentives.

To confirm construct validity, we expected medium to large positive correlations between meaningful work, work as a source of meaning and the four facets with meaningfulness in life (SoMe), socio-moral climate (SMC) and professional efficacy (MBI-GS). We hypothesized particularly large positive correlations of meaningful work and work as a source of meaning with job satisfaction and work as meaning (WAMI), thus testing concurrent validity. Regarding meaningless work, we expected large correlations with crisis of meaning in life (SoMe), general mental distress (PHQ-4), emotional exhaustion and cynicism (MBI-GS). Content validity was examined through hypothesized correlations between the ME-Work scales: The four facets of meaningful work were expected to represent a differentiated assessment of meaningful work. An aggregate score of the four facets should thus be highly (*r* > 0.70) related with the meaningful work scale, and highly negatively related with the meaningless work scale (*r* > −0.70). Moreover, the aggregate score of the four facets as well as the meaningful work scale were hypothesized to serve as preconditions for work being experienced as a source of meaning; these correlations should therefore also be large (*r* > 0.70). For the establishment of factorial validity, confirmatory factor analysis (using IBM SPSS Amos 25) was applied. In a final step, we examined the inventory’s incremental validity with regard to general mental distress and professional efficacy. We also compared the incremental predictive power of the three ME-Work modules to that of the WAMI (Steger et al., 2012). The latter inventory was chosen because it was identified, in a recent review (Both-Nwabuwe et al., 2017), as one of two sufficiently validated multidimensional measures of meaning in work. Of both, it has been used more frequently, and it has an objective comparable to the ME-Work. The second, the CMWS (Lips-Wiersma and Wright, 2012), is a complex process-oriented measure and thus not directly comparable to the ME-Work. After establishing evidence of the ME-Work’s validity, we exploratively analyzed which specific facets of meaning in work contributed to the subjective assessment of work as meaningful, meaningless or or a source of meaning. In order to identify potential gender effects, this was done for women and men separately. We thus responded to a research question that has not yet been clarified in the literature, and to a call for explicitly addressing gender differences (Bailey et al., 2019). For this purpose, six multiple regressions were conducted.

#### Participants

The inclusion criteria were a minimum age of 18 years, sufficient command of the German language, access to the Internet and current employment. Exclusion criteria were termination of the questionnaire before completion and an above-average speed of response. A total of *N* = 305 German-speaking persons completed the entire questionnaire. Only *n* = 26 of them were self-employed. Since the validity of ME-Work for self-employed persons could not be examined due to this small sample size, these cases were excluded from the sample. Of the remaining *N* = 279, one person stood out due to an extremely short response time. On closer examination, repetitive response patterns (the same response for all items of a scale) were found, so that this person was also excluded. The anomaly index showed no anomalies, so the final sample size was *N* = 278.

Mean age was 35 years, ranging from 19 to 63 years (two missing values). Seventy-five percent of the sample were female (two missing values). Thirty-five percent had completed their General Certificates of Secondary Education, 20% had an advanced-level qualification and 55% had a university degree (two missing values). Seven percent were marginally employed, 32% had a part-time and 61% a full-time job. The average weekly working hours were 34 (SD = 11). A relatively large proportion of the participants (28%) worked in the educational and social sector, 19% in the health sector, 13% in business and administration, 6% in transport and logistics, 4% each in the hotel and restaurant industry and in metal and machinery, 3% each in food, in construction, in sale, in IT and in culture. The remainder came from the sectors of nature-related professions, design and art, building service engineering, beauty, vehicles, science and research, electrical engineering, planning and construction, NGOs and physics and chemistry.

#### Measures

The literature has repeatedly shown positive relationships between meaning in work and meaning in life, satisfaction with work, socio-moral climate and work efficacy, as well as negative associations with mental distress and burnout. These measures were thus used to establish evidence of convergent and divergent construct validity. To demonstrate the advantages of ME-work, we also employed another often-used meaning in work scale for comparison. All measures are briefly described in the following.

##### The modular meaning in work inventory (ME-Work)

We employed the seven scales resulting from the pilot study described above plus two additional items to enhance the meaningful work scale. Again, all scales were rated on a six-point Likert scale ranging from 0 (strongly disagree) to 5 (strongly agree). Internal consistencies in the present study were very good, Cronbach’s alphas for meaningful work (3 items) = 0.92, for meaningless work (3 items) = 0.86, for work as a source of meaning (4 items) = 0.88, for significance (3 items) = 0.87, for coherence (3 items) = 0.85, for purpose (4 items) = 0.78 and for belonging (3 items) = 0.85. The German items as well as an English translation are available in the Supplementary Material.

##### Meaningfulness and crisis of meaning (SoMe)

These two dimensions of meaning in life are part of the Sources of Meaning and Meaning in Life Questionnaire (SoMe; Schnell and Becker, 2007; Schnell, 2009). The questionnaire’s reliability and validity have been shown in numerous studies (cf. Schnell, 2014, 2020). Meaningfulness measures the degree of experienced meaning in life, and crisis of meaning measures the degree of a perceived lack of meaning. The two constructs are relatively independent of each other, and confirmatory factor analyses have supported a two-dimensional model (Damásio et al., 2013; Schnell, 2014). Both five-item scales are rated on a six-point Likert scale ranging from 0 (strongly disagree) to 5 (strongly agree). Internal consistencies in the present study were Cronbach’s alpha = 0.79 and 0.94, respectively.

##### Job satisfaction

The general job satisfaction scale (Iwanowa, 2007) uses five items to evaluate satisfaction with various aspects of work, i.e., social relationships with superiors and colleagues, the content of work, the remuneration and work as a whole. The items are rated on a six-point Likert scale ranging from 1 (very dissatisfied) to 6 (very satisfied). According to Iwanowa (2007), the scale is a reliable and economically applicable screening instrument for an assessment of general job satisfaction. Internal consistency in the present study was Cronbach’s alpha = 0.78.

##### Socio-moral Climate Questionnaire (short)

The 21-item short version of the Socio-moral Climate Questionnaire (SMC; Verdorfer et al., 2015) measures socio-moral climate in organizations by means of five scales. These are (1) open confrontation with conflicts, (2) appreciation and respect, (3) open communication and participative cooperation, (4) assignment of responsibility and (5) organizational concern. Evidence for reliability and validity has been established (Verdorfer et al., 2015; Höge et al., 2020). Confirmatory factor analyses for the German version confirmed a second-order factor structure that justifies the aggregation of the five sub-scales into a general SMC-score (Verdorfer et al., 2015). Items are administered with a five-point Likert scale ranging from 1 (strongly disagree) to 5 (strongly agree). In the present study, internal consistency of the composite score for SMC was Cronbach’s alpha = 0.96; subscale alphas ranged from 0.76 (assignment of responsibility) to 0.91 (open confrontation with conflicts).

General mental distress was measured by the PHQ-4 (Kroenke et al., 2009), a brief four-item measure of core symptoms of depression and anxiety. It uses a four-point Likert scale ranging from 0 (not at all) to 3 (nearly every day). The PHQ-4 has demonstrated good reliability and validity in both clinical and population samples; the two-factor structure was confirmed by CFA (e.g., Löwe et al., 2010; Kerper et al., 2014). Cronbach’s alpha in the present study was 0.83.

##### Maslach Burnout Inventory – General Survey

The MBI (Maslach and Jackson, 1981; Maslach et al., 1996) is the most widely used instrument for assessing burnout. The Maslach Burnout Inventory – General Survey (MBI-GS; Schaufeli et al., 1996; German version: Cillien et al., 2006) consists of 16 items that comprise three independent scales: emotional exhaustion, cynicism and professional efficacy. The three-factor structure was confirmed in different countries and occupational groups (e.g., Langballe et al., 2006; Chirkowska-Smolak and Kleka, 2011; Mäkikangas et al., 2011). Higher values of emotional exhaustion and cynicism and lower levels of professional efficacy indicate that people might be affected by burnout. However, the three scales should not be combined into an overall value, as they represent different aspects of burnout. Several studies suggested using only those three items per scale that were characterized by low error correlations (Leiter and Shaughnessy, 2006; Leiter and Maslach, 2009). Employing the German version of the MBI-GS, the selection of these three items per dimension was statistically confirmed (Brom et al., 2015). We therefore limited ourselves to these nine items, using a seven-point Likert scale that ranged from 0 (never) to 6 (daily). Internal consistencies in the present study were Cronbach’s alpha = 0.88 for emotional exhaustion, 0.81 for cynicism and 0.71 for professional efficacy.

##### Work as Meaning Inventory

The Work as Meaning Inventory (WAMI; Steger et al., 2012) is a three dimensional questionnaire that measures meaning in work. The following three scales are included: positive meaning in work, meaning making through work and greater good motivation. The authors provided evidence for reliability, construct validity, and factorial validity in their initial study. However, the three-factor structure was not replicable or questioned in some translations of the scale (Harzer and Steger, 2012; Puchalska-Kamińska et al., 2019). We employed the German version provided by Harzer and Steger (2012) and, as suggested, only used the total score. The ten items are rated on a five-point Likert scale ranging from 1 (absolutely untrue) to 5 (absolutely true). Internal consistency in the present study was Cronbach’s alpha = 0.93 for the total score.

##### Demographics and work specification

The following demographic and work-related variables were assessed: age, gender, education, professional activity (yes/no), type of work (self-employed or employed), average weekly working hours and professional sector. Additionally, a one-item measure of challenge was included (dichotomized as 0 = adequately challenged, 1 = under- or over-challenged).

## Results

### Descriptive Statistics

Skewness and kurtosis for all ME-Work scales were in acceptable ranges (skewness < 2, kurtosis < 7; cf. Cohen et al., 2013). Table 1 shows intercorrelations, mean scores, standard deviations and internal consistencies for the seven ME-Work scales.

**TABLE 1:** Correlations between scales, means, standard deviations and reliability statistics, Study 2.

	Meaningful work	Meaningless work	Work as source of m.	Significance	Coherence	Purpose	Belonging
Meaningful work		–0.76	0.78	0.76	0.74	0.52	0.46
Meaningless work			–0.73	–0.54	–0.67	–0.48	–0.52
Work as a source of meaning				0.62	0.83	0.54	0.51
Significance					0.59	0.45	0.31
Coherence						0.47	0.52
Purpose							0.44
Total score facets	0.81	–0.71	0.81	0.79	0.83	0.77	0.71
*M*	3.92	1.01	3.35	3.62	3.48	3.47	3.86
*SD*	1.17	1.27	1.28	1.32	1.15	1.23	1.03
Cronbach’s Alpha	0.92	0.86	0.88	0.87	0.85	0.78	0.85

*N = 278. All correlations significant at p < 0.01 (two-sided).*

Relationships between ME-Work scales and age were all non-significant. Women and men did not differ significantly in any of the scales; neither did full-time employees, part-time employees and marginally employed participants. (The marginally employed reported lower values than full- and part-time employees in all scales; however, since only 7% of our sample were marginally employed, the differences did not yield statistical significance).

### Confirmatory Factor Analysis

**FIGURE 1:**
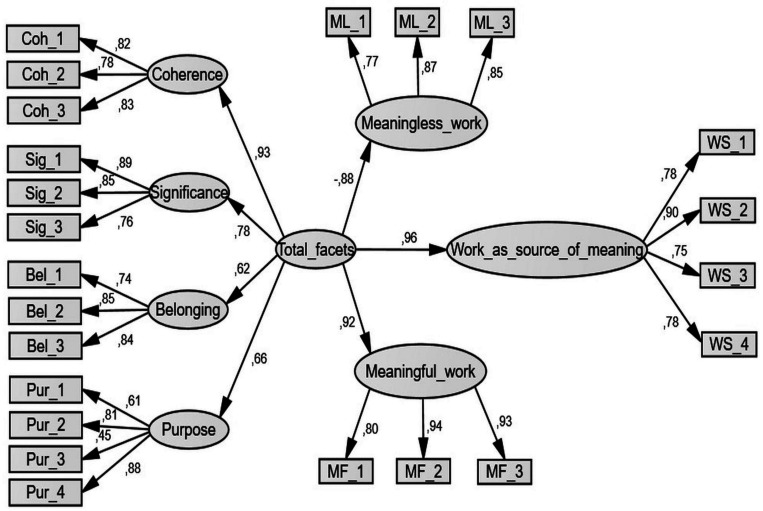
CFA of the ME-Work.

Confirmatory factor analysis (CFA) was conducted (using AMOS 25) to examine the factor structure of the ME-Work (see Figure 1). When all scales were specified as separate factors, model fit was good (χ^2^ (223) = 452.58, *p* < 0.001, SRMR = 0.050, RMSEA = 0.061, CFI = 0.950) and in line with recommended cut-off scores (CFI ≥ 0.95; SRMR ≤ 0.06; RMSEA ≤ 0.08, e.g., Hu and Bentler, 1999). No error terms were allowed to correlate. The CFA thus confirmed the modular structure of the ME-Work scales. The four facets of meaningful work served as indicators of a latent construct (parameters ranging from 0.66 to 0.93) which, in turn, predicted the three subjective experiences of meaning in work: meaningless work (-0.88), meaningful work (0.92) and work as a source of meaning (0.96).

For comparison, several other models were estimated. In one alternative model, all items of the four meaningful work facets loaded on a single factor. This model fit the data poorly, χ^2^ (227) = 1053,42, *p* < 0.001, SRMR = 0.077, RMSEA = 0.115, CFI = 0.821. Accordingly, it is not advisable to aggregate the items of all four facets to a composite score. Another model differed from the original model in that all items from the meaningless work, meaningful work and work as a source of meaning scales loaded on one single factor. Also this model had a poor fit*:*χ^2^ (225) = 622,49, *p* < 0.001, SRMR = 0.053, RMSEA = 0.080, CFI = 0.914. A third model specified meaningful and meaningless work as one factor, as had been suggested by the exploratory factor analysis outcome in Study 1, albeit with sub-optimal items measuring meaningful work. Fit for this model was also not satisfactory: χ^2^ (203) = 498,15, *p* < 0.001, SRMR = 0.052, RMSEA = 0.074, CFI = 0.932. A final model tested one single factor for all items. Fit indices for this model were very poor*:*χ^2^ (230) = 1253,30, *p* < 0.001, SRMR = 0.081, RMSEA = 0.217, CFI = 0.779. Chi-square difference tests between the suggested model and the four alternative models revealed that the suggested model fit significantly better than all four, χ^2^ (4) = 600.84, *p* < 0.001, χ^2^ (2) = 169.91, *p* < 0.001, χ^2^ (20) = 4557, *p*< 0.001 and χ^2^ (7) = 800.72, *p* < 0.001, respectively. This provides further evidence for the modular structure of the ME-Work, comprising seven factors of which four can be subsumed under a second-order factor.

### Construct Validity

Correlations between ME-Work scales and related measures were examined to assess evidence for construct validity. The following external measures were included: Meaningfulness and crisis of meaning (SoMe), job satisfaction, socio-moral climate scales (a) open confrontation with conflicts, (b) appreciation and respect, (c) open communication and participative cooperation, (d) assignment of responsibility and (e) organizational concern (SMC), general mental distress (PHQ-4), MBI-GS emotional exhaustion, cynicism and professional efficacy scales and work as meaning total score (WAMI). All predicted correlations were significant, in the expected directions and at the hypothesized level (see Table 2). Meaningful work, work as a source of meaning and all four facets established substantial positive correlations with life meaningfulness, job satisfaction, socio-moral climate, work as meaning and professional efficacy, thus offering evidence of convergent validity. They were negative related to crisis of meaning, general mental distress, emotional exhaustion and cynicism, which indicates discriminant validity. Meaningless work, on the other hand, was largely positively associated with the latter scales, as had been hypothesized. Both meaningful work and work as a source of meaning scales showed a particularly high degree of correspondence with the work as meaning total score (*r* > 0.70), indicating that the WAMI taps the same constructs as the ME-Work.

**TABLE 2:** Correlations between ME-Work Scales and related measures, Study 2.

	MW	ML	WS	Sig	Co	Pur	Bel
Meaningfulness/life	0.54	–0.47	0.55	0.53	0.58	0.32	0.39
Crisis of meaning	–0.52	0.62	–0.51	–0.38	–0.56	–0.38	–0.42
Job satisfaction	0.64	–0.69	0.70	0.44	0.68	0.65	0.66
SMC							
Total score	0.42	–0.48	0.53	0.32	0.48	0.73	0.57
Confrontation	0.36	–0.44	0.48	0.28	0.42	0.65	0.52
Respect	0.42	–0.48	0.47	0.29	0.42	0.68	0.58
Participation	0.37	–0.41	0.50	0.28	0.45	0.63	0.48
Responsibility	0.43	–0.43	0.50	0.35	0.46	0.67	0.50
Concern	0.36	–0.44	0.47	0.26	0.44	0.70	0.54
General mental distress	–0.55	0.67	–0.57	–0.37	–0.58	–0.38	–0.48
MBI-GS							
Emotional exhaustion	–0.47	0.55	–0.55	–0.31	–0.53	–0.36	–0.42
Cynicism	–0.67	0.72	–0.72	–0.53	–0.67	–0.52	–0.50
Professional efficacy	0.55	–0.49	0.52	0.44	0.55	0.34	0.35
WAMI	0.74	–0.62	0.78	0.79	0.72	0.51	0.42

*N = 278. All correlations significant at p < 0.01 (two-sided). SMC, Socio-moral climate; MBI-GS, Maslach Burnout Inventory General Survey; MW, meaningful work; ML, meaningless work; WS, work as a source of meaning; Sig, Significance; Coh, Coherence; Pur, Purpose; Bel, Belonging.*

### Incremental Validity

The ME-Work’s incremental validity was examined by analyzing its unique predictive power with regard to two critical variables: general mental distress (PHQ-4) as an indicator of negative psychological health and professional efficacy (MBI-GS) as an indicator of work performance. The unique contribution of three ME-Work modules – (1) the four facets of meaningful work, (2) meaningful and meaningless work and (3) work as a source of meaning – is shown in addition to that yielded by employing the WAMI total score (Tables 3, 4). Hierarchical multiple regressions were conducted. In an initial step for each analysis, general mental health and professional efficacy were regressed on work challenge and job satisfaction. We could thus gauge the additional amount of variance explained by various aspects of meaning in work beyond the well-researched constructs of work challenge and job satisfaction. As seen in Tables 3, 4, all ME-Work modules added significant portions of variance to the explanation of both general mental health and professional efficacy.

**TABLE 3:** Hierarchical regression analysis predicting general mental distress (PHQ-4).

	*B*	*SE B*	*CI* 95%	β	*R*	*R*^2^	*R*^2^Δ
Step 1							
Work challenge^*a*^	–0.24	0.08	[−0.39/−0.09]	−0.17**			
Job satisfaction	–0.40	0.04	[−0.48/−0.32]	−0.50***	0.59***	0.34***	
Step 2 – ME-Work 1							
Work challenge	–0.19	0.07	[−0.33/−0.05]	−0.14**			
Job satisfaction	–0.16	0.07	[-0.29/−0.04]	−0.21*			
Coherence	–0.20	0.04	[−0.29/−0.11]	−0.32***			
Significance	–0.01	0.03	[−0.08/0.05]	–0.03			
Purpose	–0.01	0.04	[−0.06/0.08]	0.02			
Belonging	–0.09	0.04	[−0.18/0.01]	−0.14*	0.65***	0.42***	0.08***
Step 2 – ME-Work 2							
Work challenge	–0.15	0.07	[−0.28/−0.01]	−0.10*			
Job satisfaction	–0.13	0.05	[−0.23/−0.03]	−0.17*			
Meaningful work	–0.03	0.04	[−0.11/0.05]	–0.05			
Meaningless work	0.27	0.04	[0.19/0.35]	0.48***	0.69***	0.48***	0.13***
Step 2 – ME-Work 3							
Work challenge	–0.22	0.07	[−0.36/−0.08]	−0.16**			
Job satisfaction	–0.22	0.05	[−0.32/−0.11]	−0.27***			
Work as source of meaning	–0.19	0.04	[−0.26/−0.11]	−0.34***	0.63***	0.40***	0.06***
Step 2 – WAMI							
Work challenge	–0.23	0.07	[−0.38/−0.09]	−0.17**			
Job satisfaction	–0.27	0.05	[−0.36/−0.18]	−0.34***			
WAMI	–0.22	0.05	[−0.31/−0.13]	−0.29***	0.63***	0.40***	0.05***

N = 278. *CI* 95% = upper level/lower level 95% confidence intervals for estimate.^*a*^0 = over- or under-challenged, 1 = adequately challenged.*p < 0.05. ** *p* < 0.01. ***p < 0.001.

**TABLE 4:** Hierarchical regression analysis predicting professional efficacy (MBI-GS).

	*B*	*SE B*	*CI* 95%	β	*R*	*R*^2^	*R*^2^Δ
Step 1							
Work challenge^*a*^	0.12	0.11	[-0.10/0.34]	0.06			
Job satisfaction	0.52	0.06	[0.40/0.64]	0.47***	0.50***	0.25***	
Step 2 – ME-Work 1							
Work challenge	0.08	0.11	[-0.13/0.28]	0.04			
Job satisfaction	0.25	0.10	[0.06/0.43]	0.22**			
Coherence	0.26	0.07	[0.14/0.39]	0.31***			
Significance	0.12	0.05	[0.03/0.21]	0.16*			
Purpose	–0.02	0.05	[-0.13/0.08]	–0.03			
Belonging	–0.01	0.06	[-0.13/0.12]	–0.01	0.59***	0.35***	0.10***
Step 2 – ME-Work 2							
Work challenge	0.06	0.11	[-0.15/0.27]	0.03			
Job satisfaction	0.24	0.08	[0.08/0.39]	0.21**			
Meaningful work	0.31	0.07	[0.18/0.44]	0.37***			
Meaningless work	–0.04	0.07	[-0.17/0.09]	–0.05	0.58***	0.34***	0.09***
Step 2 – ME-Work 3							
Work challenge	0.09	0.11	[-0.12/0.30]	0.05			
Job satisfaction	0.27	0.08	[0.11/0.43]	0.24**			
Work as source of meaning	0.26	0.05	[0.15/0.36]	0.34***	0.55***	0.31***	0.06***
Step 2 – WAMI							
Work challenge	0.11	0.11	[-0.10/0.32]	0.06			
Job satisfaction	0.35	0.07	[0.21/0.49]	0.32***			
WAMI	0.29	0.07	[0.16/0.43]	0.27***	0.55***	0.30***	0.05***

N = 278. *CI* 95% = upper level/lower level 95% confidence intervals for estimate.^*a*^0 = over- or under-challenged, 1 = adequately challenged.*p < 0.05. ** *p* < 0.01. ***p < 0.001.

### Predicting Meaningful and Meaningless Work

As the above reports point to the validity of ME-Work, we have used the inventory in a final step to explore which facets of meaning in work contributed to meaningful, meaningless and meaning-making work. For this purpose, six regression analyses were carried out (Tables 5–7). In order to detect eventual gender effects, they were calculated separately for women and men. Because fewer men than women were represented in the current data set, we applied bootstrapping in the following analyses.

**TABLE 5:** Multiple regression analysis predicting meaningful work, separated by gender.

	*B*^a^	*SE B*	*BCa CI* 90%	β	*r*_*zero order*_	*r*_*partial*_	*R*^2^
**Women**							
Coherence	0.38**	0.07	[0.29/0.48]	0.39	0.75	0.49	
Significance	0.43**	0.05	[0.30/0.54]	0.47	0.77	0.59	
Purpose	0.12*	0.05	[0.02/0.24]	0.12	0.52	0.21	
Belonging	0.07	0.06	[−0.00/0.15]	0.06	0.43	0.11	0.75***
**Men**							
Coherence	0.32*	0.12	[0.16/0.45]	0.27	0.73	0.34	
Significance	0.44**	0.08	[0.34/0.53]	0.53	0.79	0.62	
Purpose	0.05	0.09	[−0.08/0.15]	0.05	0.56	0.07	
Belonging	0.20	0.12	[−0.06/0.50]	0.16	0.57	0.23	0.74***

N = 278. *BCa CI* 90% = upper level/lower level 90% bias-corrected and accelerated (BCa) bootstrap interval.^*a*^Based on 1000 bootstrap samples. *p < 0.05. **p < 0.01. ***p < 0.001 (one-sided).

About three fourth of the variance in meaningful work was explained by the four facets of meaning in work. The strongest predictors among women were significance and coherence, followed by purpose. Among men, significance explained the largest amount of variance in meaningful work, followed by coherence (see Table 5).

Women and men differed more profoundly with regard to the predictors of work experienced as meaningless (Table 6). Altogether, more than half of the variance of meaningless work was explained by the four facets of meaning in work. For men, a lack of coherence was the main predictor, while a lack of significance and purpose added further explanation. Also for women, a lack of coherence was the strongest predictor. A lack of belonging explained another substantial amount of variance, followed by a lack of significance and a lack of purpose.

**TABLE 6:** Multiple regression analysis predicting meaningless work, separated by gender.

	*B*^a^	*SE B*	*BCa CI* 90%	β	*r*_*zero order*_	*r*_*partial*_	*R*^2^
**Women**							
Coherence	−0.42***	0.10	[-0.66/-0.17]	–0.39	-0.67	-0.38	
Significance	−0.18*	0.08	[-0.27/-0.09]	–0.18	-0.53	-0.21	
Purpose	−0.13*	0.07	[-0.22/-0.04]	–0.13	-0.46	-0.16	
Belonging	−0.26**	0.08	[-0.38/-0.17]	–0.22	-0.52	-0.26	0.54***
**Men**							
Coherence	−0.60***	0.18	[-0.95/-0.16]	–0.48	-0.70	-0.43	
Significance	−0.18*	0.08	[-0.27/-0.09]	–0.18	-0.57	-0.20	
Purpose	−0.13*	0.07	[-0.22/-0.04]	–0.12	-0.53	-0.13	
Belonging	–0.10	0.18	[-0.53/0.27]	–0.07	-0.49	-0.08	0.53***

N = 278. *BCa CI* 90% = upper level/lower level 90% bias-corrected and accelerated (BCa) bootstrap interval. ^*a*^Based on 1000 bootstrap samples. *p < 0.05. **p < 0.01. ***p < 0.001 (one-sided).

The four facets of meaning in work also explained about three fourth of the variance in work as a source of meaning. For both men and women, coherence showed by far the highest regression weight. Purpose and significance were significant predictors too (Table 7).

**TABLE 7:** Multiple regression analysis predicting work as a source of meaning, separated by gender.

	*B*^a^	*SE B*	*BCa CI* 90%	β	*r*_*zero order*_	*r*_*partial*_	*R*^2^
**Women**							
Coherence	0.68***	0.06	[0.54/0.81]	0.62	0.83	0.65	
Significance	0.16**	0.05	[0.09/0.23]	0.15	0.61	0.23	
Purpose	0.15*	0.06	[0.08/0.22]	0.14	0.52	0.22	
Belonging	0.12	0.08	[0.02/0.21]	0.10	0.51	0.15	0.73***
**Men**							
Coherence	0.75***	0.11	[0.61/0.86]	0.61	0.85	0.65	
Significance	0.17**	0.06	[0.05/0.30]	0.20	0.67	0.30	
Purpose	0.19*	0.08	[-0.01/0.37]	0.19	0.64	0.28	
Belonging	0.00	0.10	[-0.23/0.29]	0.00	0.55	0.01	0.77***

N = 278. *BCa CI* 90% = upper level/lower level 90% bias-corrected and accelerated (BCa) bootstrap interval.^∗^*p* < 0.05. ** *p* < 0.01. ***p < 0.001 (one-sided). ^*a*^Based on 1000 bootstrap samples.

### Qualitative Substantiation

In order to relate the quantitatively validated ME-Work constructs to lived experiences and thus test and underpin them, three semi-structured interviews were conducted. The interviewees were a bus driver, a ski instructor and an actor who worked full-time in these professions. The interviews started with introductory questions about daily work tasks, working conditions, payment, and why they had chosen the job. This was followed by questions on what the interviewees experienced as meaningful and what as meaningless in their jobs, on significance, purpose, coherence and belonging as well as on opportunities for growth and self-realization in their jobs.

In all three, work perceived as meaningful went hand in hand with the subjective experience that this activity has a *significance* for others. As the bus driver explained: “You connect city districts. It gives older people freedom, because they can go shopping by bus and go to the market and back home again. I take people to work, children to school.” The ski instructor referred to his personal contribution to showing ski students “that they can achieve something” and the actor “hopes to touch people with one’s work in some way and also to come across something that speaks to them and they can take home with them.”

*Purpose* is experienced when a person can identify with the values and goals that are aspired to and lived in the respective workplace (e.g., sustainability, profit, innovation, stability.). If this is the case, people can work in unison and individuals take responsibility for the whole. The ski instructor: “Team spirit is the greatest value we try to convey –also to the outside world, so that we as a ski school form a unit.” The actor emphasized the need for fairness: “It starts with the contracts, that they are fair for everyone – i.e., lighting technicians, make-up, costumes etc. – and that’s why the atmosphere is good.” However, if such an orientation is missing, if it is ’only a facade’ or obviously puts people at the back, cynicism and loss of meaning are not far off. The bus driver talked about stressful working conditions resulting from the company’s orientation: “The driving personnel is a ‘cost factor’. The management does not take any measures and the next bus driver is sure to come [waiting to be employed]. In the year in which I started working, at least four people died. Heart attack.”

On the other hand, he said that among his colleagues “things are pretty good, fortunately. You always meet when you have a break. Then you can let go of some of the things that keep you so busy and happen on the tour.” He thus described a sense of *belonging*, of being part of a larger whole. Although the management seemed not to provide it, he found it in sharing experiences with his colleagues. The ski instructor stated: “I get the most meaning from positive feedback. When you are told that you have done well.” Reliable appreciation is a core element of a work climate enabling belonging (Schnell et al., 2019). The actor experienced belonging when he felt that the employer had a real interest in the work: “A good employer often appears at the rehearsals or during the shoot and looks at what is being produced artistically there, and sympathizes.”

*Coherence* of professional activity is reflected, among other things, in the fit between person and job (“work-role fit”). For the actor, this was not in question: “Well, I couldn’t imagine doing anything else, even now after 40–50 years, because I need the work like I need water to drink.” Coherence also depends on how well professionals know themselves, their strengths and weaknesses, values and interests, and how these insights can be put into practice later on. The ski instructor showed good self-knowledge – which does not automatically result in high coherence: “I am a quite patient person, so teaching in itself and passing on knowledge to others suits me well, but the leadership position [which he held] does not fit me one hundred percent.” The bus driver had all the necessary skills, as he knew; nevertheless, he felt that there was more to him than that: “If you take a certain direction, it will continue in that direction, even if things change. But if it is right for me? I dare to doubt that, more or less. It is a necessity. It was and is a necessity.”

For the three interviewees, the perception of their *work as meaningful* was related to the four criteria of significance, coherence, purpose and belonging. And yet this seems to be not merely a question of the highest possible degree but also of ensuring that contradictions and discrepancies do not become too great. So the bus driver explained: “It’s 180 hours a month that I work. And, of course, you can’t say that it all sucks. You see a lot of people. People come up to you. There are grotesque situations, all kinds of situations. Even in conversation with each other. Be it of a polite nature or more aggressive. That’s all right. I can say ’yes’ to that.” The ski instructor related: “People come to us with a certain goal. They want to learn to ski or snowboard and ideally we can offer this service and achieve a certain success. From that point of view, it is meaningful to me.” The actor too saw meaning in his professional activity – even more than that: for him, his work was a *source of meaning*: “I didn’t do this to earn money, this profession. It was solely an inner voice that said ’I want to do this job’. It also gives me strength for life, also in my private life.” He had the chance to grow and self-actualize in his work.

Of the three respondents, only the bus driver reported experiences of *meaninglessness*. He criticized working conditions as inappropriate and meaningless. Nevertheless, this meaninglessness was apparently outweighed by his general affirmation of the job (“I can say ‘yes’ to that.”), and he still saw a point in taking responsibility: “I don’t give a damn what the timetable is like. I’m doing my best, and I’m driving the way the old woman in the back needs it right now, who hasn’t found a seat and is holding on to the bar. Yes, because she doesn’t stand a chance if you have to brake. Splat, she’s gone like I don’t know what. She’s flying.”

## Discussion

The present article introduced ME-Work, a new inventory for measuring several aspects of meaning in work. The ME-Work comprises three modules: Module one operationalizes – based on the current state of empirical research on meaning in life – four facets of meaning in work, namely coherence, significance, purpose and belonging. Module two refers to a subjective assessment of one’s own work as meaningful or meaningless, while module three captures the significance of this work for one’s own meaning in life, as a source of meaning. In the first part of the article the process of item development and the results of a pilot study were presented. The internal consistency of the scales proved to be very satisfactory and suggested a reduction of the scales to three or four items. Two exploratory factor analyses showed the theoretically expected factors, although meaningful and meaningless work items loaded on one factor only. This was attributed to the fact that one item of the meaningful work scale loaded differently than expected and another item showed an insufficient loading. We therefore generated two additional items to ensure that the construct was adequately captured in the validation study.

The instrument tested in the validation study then contained seven scales and a total of 23 items (see Supplementary Material). As in the pilot study, the scales’ internal consistencies proved to be very good. The scales intercorrelated in the expected way, which confirmed the modular structure of the inventory: an aggregate of the four facets of meaning in work in module one showed over 65% overlap with both meaningful work and work as a source of meaning, ensuring that the use of modules two or three alone taps the same phenomena as module one. This confirms the validity of the intended modular nature. Modules two and/or three can be used for screening purposes, e.g., in regular organizational employee surveys. Despite their brevity, they tap key experiences. Module two informs about people’s perception of their work as meaningful or meaningless. This evaluation addresses a basic and fundamental question which should theoretically be answerable positively with regard to all professions that meet the original – and never explicitly questioned – understanding of work: activities and labor necessary to the survival of society (The Editors of Encyclopaedia Britannica, 2018). However, a large number of today’s companies pursue objectives that contradict this idea to a greater or lesser extent, thus impeding experiences of meaningful work from the outset (Graeber, 2018). A separate assessment of meaningfulness and meaninglessness connects to a large body of research demonstrating that positive and negative experiences are not just opposite ends of one continuum. They can co-occur (as the bus driver reports in the chapter on qualitative substantiation), develop differently and show differential relationships with other constructs (Diener and Emmons, 1984; Chamberlain, 1988; regarding meaning in life: Scannell et al., 2002; Schnell, 2009). In studies on meaning in work, meaninglessness is a strongly under-researched topic (Bailey and Madden, 2019), certainly also because of a lack of respective scales. Module three goes beyond the meaningfulness of a specific job and highlights its role for a person’s meaning in life. This assessment might be particularly relevant when identification with work is of interest, either in a positive sense or with regard to boundarylessness and self-exploitation, boundarylessness and self-exploitation (cf. Lips-Wiersma and Mcmorland, 2006; Dempsey and Sanders, 2010). Additionally, or independently, module one can be used when a differentiated insight into the underlying mechanisms of evaluating meaning in work is desired. Based on this, HR managers can develop customized applications.

A CFA with a good fit also confirmed the modular structure of ME-Work. Four latent factors – significance, coherence, purpose and belonging – represented module one; two latent factors – meaningful and meaningless work – represented module two; one latent factor – work as a source of meaning – represented module three. The conceptualization of meaningful and meaningless work as two-dimensional yielded a better model fit than a one-dimensional conceptualization (as had been suggested by the exploratory factor analysis in the pilot study), most likely due to the replacement of two meaningful work items after the pilot study.

Several variables were taken into account for construct validation. All correlations showed up as expected. The meaningful work, work as a source of meaning and significance scales showed high overlap with the WAMI total score. This reflects the fact that these constructs are also targeted by the WAMI – even if they were not distinguishable by factor analysis in the version used (Harzer and Steger, 2012). However, there were further substantial overlaps with purpose and belonging, which are not explicitly addressed in the WAMI. In contrast to the WAMI, the ME-Work shows a higher degree of differentiation, which is closely linked to theoretical and empirical findings in the literature on meaning in life and meaning in work.

Meaningful work correlated substantially with meaningfulness in life, job satisfaction, socio-moral climate and professional efficacy. This was also true for the scale work as a source of meaning, which, however, showed even closer links to job satisfaction, but also to the facets of socio-moral climate. The latter finding suggests that openness, participation and the allocation of responsibility at work are particularly conducive to the realization and development of personal values and allow personal growth.

The high correlations between meaningful work and work as a source of meaning on the one hand and meaningfulness in life on the other point to a spill-over effect: People who experience their profession as meaningful might evaluate their entire life as more meaningful. Johnson and Jiang (2017) were able to prove in their two-wave study that meaningful work actually predicted later “work-to-life enrichment.” People who experienced their job as meaningful stated three months later that their job gave them the energy to carry out important activities in their daily lives, that their job improved their mood at home and was generally beneficial in coping with everyday life.

Meaningful work, meaningless work and work as a source of meaning also showed the expected correlations with crisis of meaning, general mental distress, emotional exhaustion and cynicism. Especially the high correlation of the ME-Work scales with cynicism, a core component of burnout, stood out. Cynicism thus appears as an opposite of experienced meaning in work, which confirms earlier studies that concluded that a lack of meaning can quickly turn into inner distance and cynicism (Holbeche and Springett, 2009).

The close links between the ME-Work scales and general mental distress once again confirmed the importance that experiencing meaning in work has for mental health (Allan et al., 2019; Lease et al., 2019). With regard to the four facets of meaning, mental distress was particularly strongly associated with low coherence and low belonging. A closer look at the correlations of the four facets with neighboring scales revealed differential relationships with socio-moral climate. This measure of a perceived democratic, participatory corporate culture is reflected in particularly high correlations (*r* > 0.60) with the facet purpose. This suggests that the perception of a socio-moral climate might also derive from socially responsible action by the organization, transcending profit-related goals. Moreover, the feeling of belonging correlated highly with socio-moral climate (*r* > 0.50), thus emphasising that such an appreciative-participative climate is actually associated with perceived affinity and loyalty.

An examination of the incremental validity of the ME-Work scales demonstrated that the addition of the ME-Work modules substantially explained further variance beyond the known work-relevant characteristics of work challenge and job satisfaction. The meaningless work scale proved to be particularly significant in predicting mental stress, thus offering a good indication of general mental distress. When the ME-Work scales were employed to predict professional efficacy, i.e., satisfaction with one’s work achievements and expectations of continued effectiveness at work, the scales coherence, meaningful work and work as a source of meaning proved to be important predictors beyond work challenge and job satisfaction. Once again this shows that the experience of meaning in work differs from satisfaction with work. This becomes even more evident when we consider that a lot of reported job satisfaction actually is “resigned satisfaction” (Unterrainer et al., 2013). It represents an acceptance of the circumstances, based on decreased levels of aspiration. According to Unterrainer et al. (2013), in a review of respective studies the proportion of resigned satisfied employees amounted to 25–45%. As would be expected, this type of job satisfaction is also associated with low levels of meaningful work (Schnell, 2018).

Finally, this article has explored the extent to which the four facets of meaning in work each contributed to the prediction of meaningful work, meaningless work and work as a source of meaning. Significance was particularly important for the prediction of work experienced as meaningful. This ties in well with findings about the motivation created by knowledge about the significance of one’s work (Grant, 2008; Allan, 2017). Purpose also played a significant, albeit smaller role. Since it is likely that many employees are not even aware of their organizations’ real purpose (Bhattacharya et al., 2008), the effect that purpose can have on experiencing work as meaningful might be understated in the present study. Research that focuses on organizations with a clear and powerful purpose and compares them with others will be helpful to test this hypothesis.

Interestingly, belonging only served as a substantial predictor of experiences of meaning in work in the case of its lack, and only among women. For them, a missing sense of cohesion and loyalty was associated with the experience of their work being meaningless. This finding might be explained by the theory that women care more than men about interpersonal relationships, which has received considerable support (Yang and Girgus, 2019). In their meta-analytic review, Yang and Girgus (2019) also found evidence for substantially higher sociotropy among women, defined as the tendency to overemphasize maintaining positive social relationships. This effect was stronger in individualistic than in collectivistic countries, where interpersonal harmony and collaboration are valued for and by both genders. We might therefore hypothesize that the facet belonging will take on a different, more prominent role in countries with a collectivist orientation – an assumption to be examined in further studies.

Coherence, i.e., the fit of a job to a person’s characteristics, skills and general life scheme, contributed substantially to the explanation of all three experiences of meaning in work. This was the case for men and women alike. For work to be experienced as a source of meaning, coherence showed up as the most important criterion. It was thus not necessarily a work that “saves the world,” a work of obvious prosocial or life-changing value. Rather, it seems to be a sense of having found something that is “entirely mine” – that can be “owned” by me, i.e., another term for authenticity (Pedersen, 2018; Schnell, 2020). This might be of particular importance in Western societies characterized by multi-optionality (Maas and Bühler, 2015) and functional differentiation (Luhmann, 1977), where processes do not follow a superordinate worldview. Individuals must determine their position in multiple subsystems (work, family, politics, religion, consumption, leisure, etc.), and it is their responsibility to ensure that these positions are coherent with each other. Creating coherence is a challenge for everyday life, but also for professional life, given that a large number of people perceive low or no fit between themselves and their work (Däfler and Dannhäuser, 2016; Schnell, 2018). This finding thus calls for renewed efforts to increase our knowledge about how to put the right people in the right jobs – beyond criteria of performance and over- or underchallenge. Approaches like job crafting, i.e., employee-initiated changes, seem to be promising in this regard (Tims et al., 2016; Lysova et al., 2019).

Our results all point in the same direction: The more meaningful the experience of their professional life, the better off professionals are. Nevertheless, there are various indications that meaningful work represents – or can represent – a double-edged sword (Bailey et al., 2019; Schnell et al., 2019; Schnell, 2020). Thus, Dempsey and Sanders (2010) were able to show that work that serves as a source of meaning can go hand in hand with self-exploitation. Hu and Hirsh (2017) demonstrated that in many cases people are prepared to accept lower pay in favor of meaningful work. Still, all relations reported in the present article were linear correlations. (We have tested other functions but found no evidence of U-shaped relationships). This suggests that the above-mentioned negative effects cannot be explained by “too much meaning” alone. Future research should focus its attention on the identification of factors that do not necessarily cancel out experiences of meaning, but are nevertheless detrimental to employees’ quality of life, such as, e.g., their economic situation, personal aspirations, instrumentalization of meaning or purpose by employers, etc. Furthermore, adequate self-care plays an equally important role in professional life as elsewhere (Santana and Fouad, 2017) and should be practiced by employees and supported by employers.

### Limitations

Some limitations of our study should be acknowledged. First, the study used a cross-sectional design and self-reported measures; common method bias might thus inflate the observed relationships between variables. Although a separation of measurements over time had been planned (Podsakoff et al., 2003), this was made impossible by the outbreak of the COVID-19 pandemic. Any data that would be collected in this context would not be comparable with the previously collected data. Second, while our sample included a broad range of employment types and branches, it cannot be viewed as representative. However, the findings reported here are supported in their generalizability (Joshanloo, 2014) by the fact that the ME-Work has also proven reliable and valid in an Italian context (Tommasi et al., under review). Third, although targeted, we did not succeed in validating the ME-Work scales for self-employed individuals. This should be the aim of further research, especially since the establishment of one-person enterprises has been strongly promoted in countries like Austria (Bögenhold and Klinglmair, 2017) and Germany (Keck, 2016), which poses many challenges with regard to meaningful work.

## Conclusion

The ME-Work inventory is an economic and reliable instrument for the differentiated measurement of meaning in work. Evidence for construct, factorial and incremental validity is provided by the present article. Due to the availability of three modules, the use of the inventory can be adapted to the current needs of researchers or practitioners. With only six items (Module two) it is possible to screen both for subjective evaluation of one’s work as meaningful and as meaningless, thus giving access to two relatively independent dimensions of experience. Module three can be employed to assess a strong identification with and passion for one’s work, which might also be relevant with regard to the dangers of boundaryless work. Module one enables a fine-grained analysis of four facets, or mechanisms, of meaning in work. This can be especially fruitful for practical use with individuals who approach superiors, consultants or coaches with questions of meaning related to their jobs.

The findings reported here also offer some noteworthy insights. For a start, perceived coherence plays a particularly crucial role in the experience of meaningful work. To optimize the fit between person and professional activity can thus be described as the primary task of managers when it comes to enabling meaningful work. In doing so, the person as a whole should be in the foreground, including his or her characteristics, values and life plans. A limitation to strengths and skills alone is not conducive to achieving the desired results. In addition, our data strongly suggest that meaningless work is a risk factor for mental well-being. With regard to the facets of meaning in work, low coherence and low belonging stood out here. We can therefore conclude that meaning in work is not only a positive factor, but that the experience of meaninglessness at work is associated with psychological suffering. Ignoring the question of meaning at the workplace can therefore have serious consequences for employers and employees alike.

## Data Availability Statement

The raw data supporting the conclusions of this article will be made available by the authors, without undue reservation.

## Ethics Statement

The studies involving human participants were reviewed and approved by Review Board (Psychology) of the University of Innsbruck. Written informed consent for participation was not required for this study in accordance with the national legislation and the institutional requirements.

## Author Contributions

TS: conceptualization, methodology, formal analysis, writing – original draft, and project administration. CH: conceptualization, investigation, methodology, and writing – reviewing and editing. Both authors contributed to the article and approved the submitted version.

## Conflict of Interest

The authors declare that the research was conducted in the absence of any commercial or financial relationships that could be construed as a potential conflict of interest.

## References

[B1] Allan B. A. (2017). Task significance and meaningful work: a longitudinal study. *J. Vocat. Behav.* 102 174–182. 10.1016/j.jvb.2017.07.011

[B2] Allan B. A. Batz-Barbarich C. Sterling H. M. Tay L. (2019). Outcomes of meaningful work: a meta-analysis. *J. Manag. Stud.* 56 500–528. 10.1111/joms.12406

[B3] Allan B. A. Dexter C. Kinsey R. Parker S. (2018). Meaningful work and mental health: job satisfaction as a moderator. *J. Ment. Health* 27 38–44. 10.1080/09638237.2016.1244718 27841056

[B4] Arnoux-Nicolas C. Sovet L. Lhotellier L. Di Fabio A. Bernaud J. L. (2016). Perceived work conditions and turnover intentions: the mediating role of meaning of work. *Front. Psychol.* 7:704. 10.3389/fpsyg.2016.00704 27242616 PMC4863887

[B5] Aßländer M. S. (2005). *Bedeutungswandel der Arbeit. Versuch einer historischen Rekonstruktion (Bd. 40).* Reihe Aktuelle Analysen München: Hanns-Seidel-Stiftung.

[B6] Bailey C. Madden A. (2016). What makes work meaningful–or meaningless? *MIT Sloan Manag. Rev.* 57 52–63.

[B7] Bailey C. Madden A. (2017). Time reclaimed: temporality and the experience of meaningful work. *Work Employ. Soc.* 31 3–18. 10.1177/0950017015604100

[B8] Bailey C. Madden A. (2019). “We’re not scum, we’re human”: agential responses in the face of meaningless work. *Scand. J. Manag.* 35:101064 10.1016/j.scaman.2019.101064

[B9] Bailey C. Madden A. (2020). “Contemporary challenges in meaningful work,” in *The Future of Work and Employment*, eds Wilkinson A. Barry M. (Cheltenham: Edward Elgar Publishing), 65–82. 10.4337/9781786438256.00012

[B10] Bailey C. Madden A. Alfes K. Shantz A. Soane E. (2017). The mismanaged soul: existential labor and the erosion of meaningful work. *Hum. Resour. Manag. Rev.* 27 416–430. 10.1016/j.hrmr.2016.11.001

[B11] Bailey C. Yeoman R. Madden A. Thompson M. Kerridge G. (2019). A review of the empirical literature on meaningful work: progress and research agenda. *Hum. Resource Dev. Rev.* 18 83–113. 10.1177/1534484318804653

[B12] Bal P. M. Dóci E. (2018). Neoliberal ideology in work and organizational psychology. *Eur. J. Work Organ. Psychol.* 27 536–548. 10.1080/1359432X.2018.1449108

[B13] Bhattacharya C. B. Sen S. Korschun D. (2008). Using corporate social responsibility to win the war for talent. *MIT Sloan Manag. Rev.* 49 37–44.

[B14] Bögenhold D. Klinglmair A. (2017). One-person enterprises and the phenomenon of hybrid self-employment: evidence from an empirical study. *Empirica* 44 383–404. 10.1007/s10663-016-9332-8

[B15] Both-Nwabuwe J. Dijkstra M. Beersma B. (2017). Sweeping the floor or putting a man on the moon: how to define and measure meaningful work. *Front. Psychol.* 8:1658. 10.3389/fpsyg.2017.01658 PMC5626826 29033867

[B16] Brom S. S. Buruck G. Horváth I. Richter P. Leiter M. P. (2015). Areas of worklife as predictors of occupational health–a validation study in two German samples. *Burn. Res.* 2 60–70. 10.1016/j.burn.2015.05.001

[B17] Bühner M. (2011). *Einführung in die Test- und Fragebogenkonstruktion.* München: Pearson.

[B18] Bunderson J. S. Thompson J. A. (2009). The call of the wild: zookeepers, callings, and the double-edged sword of deeply meaningful work. *Adm. Sci. Q.* 54 32–57. 10.2189/asqu.2009.54.1.32 21821037

[B19] Cardador M. T. Caza B. B. (2012). Relational and identity perspectives on healthy versus unhealthy pursuit of callings. *J. Career Assess.* 20 338–353. 10.1177/1069072711436162

[B20] Chamberlain K. (1988). On the structure of subjective wellbeing. *Soc. Indic. Res.* 20 581–604.

[B21] Chirkowska-Smolak T. Kleka P. (2011). The Maslach Burnout Inventory–General Survey: validation across different occupational groups in Poland. *Polish Psychol. Bull.* 42 86–94. 10.2478/v10059-011-0014-x

[B22] Cillien P. Fischbach A. Mörsdorf A. Scherp E. Schaufeli W. B. (2006). *Maslach Burnout Inventory–General Survey Deutsche Version 1.0 (MBI-GS-D V1. 0).* Unpublished manuscript.

[B23] Cohen J. Cohen P. West S. G. Aiken L. S. (2013). *Applied Multiple Regression/Correlation Analysis for the Behavioral Sciences.* Abingdon: Routledge.

[B24] Crane A. Matten D. Spence L. (2019). *Corporate Social Responsibility: Readings and Cases in a Global Context.* Abingdon: Routledge.

[B25] Crowley M. Hodson R. (2014). Neoliberalism at work. *Soc. Curr.* 1 91–108.

[B26] Däfler M. Dannhäuser R. (2016). *Glücklich im Beruf.* Available online at: https://www.ist-hochschule.de/blog/studie-gluecklich-im-beruf-veroeffentlicht/ (accessed July 24, 2020).

[B27] Damásio B. F. Koller S. H. Schnell T. (2013). Sources of Meaning and Meaning in Life questionnaire (SoMe): psychometric properties and sociodemographic findings in a large Brazilian sample. *Acta de Investig. Psicol.* 3 1205–1227. 10.1016/S2007-4719(13)70961-X

[B29] Daniel J. L. (2015). Workplace spirituality and stress: evidence from Mexico and US. *Manag. Res. Rev.* 38 29–43. 10.1108/MRR-07-2013-0169

[B28] Dempsey S. E. Sanders M. L. (2010). Meaningful work? Nonprofit marketization and work/life imbalance in popular autobiographies of social entrepreneurship. *Organization* 17 437–459. 10.1177/1350508410364198

[B30] Dich N. Lund R. Hansen ÅM. Rod N. H. (2019). Mental and physical health effects of meaningful work and rewarding family responsibilities. *PLoS One* 14:e0214916. 10.1371/journal.pone.0214916 31017925 PMC6481914

[B31] Diener E. Emmons R. A. (1984). The independence of positive and negative affect. *J. Pers. Soc. Psychol.* 47 1105–1117. 10.1037/0022-3514.47.5.1105 6520704

[B32] Duffy R. D. Dik B. J. (2013). Research on calling: what have we learned and where are we going? *J. Vocat. Behav.* 83 428–436. 10.1016/j.jvb.2013.06.006

[B33] Duffy R. D. Allan B. A. Autin K. L. Bott E. M. (2013). Calling and life satisfaction: it’s not about having it, it’s about living it. *J. Couns. Psychol.* 60 42–52. 10.1037/a0030635 23163611

[B34] Duffy R. D. Allan B. A. Autin K. L. Douglass R. P. (2014). Living a calling and work well-being: a longitudinal study. *J. Couns. Psychol.* 61 605–615. 10.1037/cou0000042 25181588

[B35] Geldenhuys M. Taba K. Venter C. M. (2014). Meaningful work, work engagement and organizational commitment. *SA J. Ind. Psychol.* 40:1098 10.4102/sajip.v40i1.1098

[B36] George L. S. Park C. L. (2016). Meaning in life as comprehension, purpose, and mattering: toward integration and new research questions. *Rev. Gen. Psychol.* 20 205–220. 10.1037/gpr0000077

[B37] Glaser J. Hornung S. Höge T. Seubert C. (2017). “Self-actualization in modern workplaces. Time-lagged effects of new job demands and job resources on motivation, meaning and self-efficacy at work,” in *Advances in Social and Occupational Ergonomics*, ed. Goossens R. H. M. (Cham: Springer), 253–263. 10.1007/978-3-319-60828-0_26

[B38] Glavas A. Kelley K. (2014). The effects of perceived corporate social responsibility on employee attitudes. *Bus. Ethics Q.* 24 165–202. 10.5840/beq20143206

[B39] Graeber D. (2018). *Bullshit Jobs–A Theory.* London: Allen Lane.

[B40] Grant A. M. (2008). The significance of task significance: job performance effects, relational mechanisms, and boundary conditions. *J. Appl. Psychol.* 93 108–124. 10.1037/0021-9010.93.1.108 18211139

[B41] Harzer C. Steger M. F. (2012). *Meaning at work: the german adaptation of the Work and Meaning Inventory (WAMI).* Moscow: Poster at ECPP.

[B42] Höge T. Schnell T. (2012). Kein Arbeitsengagement ohne Sinnerfüllung. Eine Studie zum Zusammenhang von work engagement, Sinnerfüllung und Tätigkeitsmerkmalen. *Wirtschaftspsychologie* 1 91–99.

[B43] Höge T. Strecker C. Hausler M. Huber A. Höfer S. (2020). Perceived socio-moral climate and the applicability of signature character strengths at work: a study among hospital physicians. *Appl. Res. Q. Life* 15 463–484. 10.1007/s11482-018-9697-x PMC7116458 33304415

[B44] Höge T. Weber W. G. (2018). “Das soziomoralische Organisationsklima und Sinnerfüllung in der Arbeit: Erkenntnisse über zwei Gesundheitsressourcen,” in *Fehlzeiten-Report 2018*, eds Badura B. Ducki A. Schröder H. Klose J. Meyer M. (Berlin: Springer), 225–233. 10.1007/978-3-662-57388-4_19

[B45] Hackman R. Oldham G. R. (1975). Development of the job diagnostic survey. *J. Appl. Psychol.* 60 159–170. 10.1037/h0076546

[B46] Holbeche L. Springett N. (2009). *In Search of Meaning at Work.* UK: Roffey Park Institute.

[B47] Hu J. Hirsh J. B. (2017). Accepting lower salaries for meaningful work. *Front. Psychol.* 8:1649. 10.3389/fpsyg.2017.01649 29085310 PMC5649195

[B48] Hu L. T. Bentler P. M. (1999). Cutoff criteria for fit indexes in covariance structure analysis: conventional criteria versus new alternatives. *Struct. Equ. Modeling* 6 1–55. 10.1080/10705519909540118

[B49] Hulshof I. L. Demerouti E. Le Blanc P. M. (2020). Day-level job crafting and service-oriented task performance: the mediating role of meaningful work and work engagement. *Career Dev. Intl.* 10.1108/CDI-05-2019-0111 [ahead-of-print]

[B50] Iwanowa A. (2007). “Formen der Arbeitszufriedenheit (faz) – Ergebnisse der Überprüfung von Gütekriterien des Kurzfragebogens,” in *Arbeit und Gesundheit*, eds Richter P. Rau R. Mühlpfordt S. (Lengerich: Pabst Science Publishers), 110–129.

[B52] Jaeggi R. (2014). *Alienation.* New York, NY: Columbia University Press.

[B51] Jahoda M. (1982). *Employment and Unemployment.* Cambridge: Cambridge University Press.

[B53] Johnson M. J. Jiang L. (2017). Reaping the benefits of meaningful work: the mediating versus moderating role of work engagement. *Stress Health* 33 288–297. 10.1002/smi.2710 27647548

[B54] Joshanloo M. (2014). Eastern conceptualizations of happiness: fundamental differences in Eastern and Western views. *J. Happiness Stud.* 15 475–493. 10.1007/s10902-013-9431-1

[B55] Keck W. (2016). Do we really think small first? How to go further with CSR on micro enterprises. *uwf UmweltWirtschaftsForum* 24 361–367. 10.1007/s00550-016-0424-5

[B56] Kerper L. Spies C. Tillinger J. Wegscheider K. Salz A. L. Weiss-Gerlach E. (2014). Screening for depression, anxiety and general psychological distress in preoperative surgical patients: a psychometric analysis of the Patient Health Questionnaire 4 (PHQ-4). *Clin. Health Promot.* 4 5–14. 10.29102/clinhp.14002

[B57] Koo T. K. Li M. Y. (2016). A guideline of selecting and reporting intraclass correlation coefficients for reliability research. *J. Chiropr. Med.* 15 155–163. 10.1016/j.jcm.2016.02.012 27330520 PMC4913118

[B58] Kroenke K. Spitzer R. L. Williams J. B. Monahan P. O. Loewe B. (2009). An ultra-brief screening scale for anxiety and depression: the PHQ–4. *Psychosomatics* 50 613–621. 10.1016/S0033-3182(09)70864-3 19996233

[B59] Krug G. Drasch K. Jungbauer-Gans M. (2019). The social stigma of unemployment: consequences of stigma consciousness on job search attitudes, behaviour and success. *J. Labour Mark. Res.* 53:11 10.1186/s12651-019-0261-4

[B60] Lambert N. M. Stillman T. F. Hicks J. A. Kamble S. Baumeister R. F. Fincham F. D. (2013). To belong is to matter: sense of belonging enhances meaning in life. *Pers. Soc. Psychol. Bull.* 39 1418–1427. 10.1177/0146167213499186 23950557

[B61] Langballe E. M. Falkum E. Innstrand S. T. Aasland O. G. (2006). The factorial validity of the Maslach Burnout Inventory–General Survey in representative samples of eight different occupational groups. *J. Career Assess.* 14 370–384. 10.1177/1069072706286497

[B62] Lease S. H. Ingram C. L. Brown E. L. (2019). Stress and health outcomes: do meaningful work and physical activity help? *J. Career Dev.* 46 251–264. 10.1177/0894845317741370

[B63] Leiter M. P. Maslach C. (2009). Nurse turnover: the mediating role of burnout. *J. Nursing Manag.* 17 331–339. 10.1111/j.1365-2834.2009.01004.x10.1111/j.1365-2834.2009.01004.x 19426369

[B64] Leiter M. P. Shaughnessy K. (2006). The areas of worklife model of burnout: tests of mediation relationships. *Ergon. Intl. J.* 28 327–341.

[B65] Leontiev A. N. (1982). *Tätigkeit, Bewusstsein, Persönlichkeit.* Köln: Pahl-Rugenstein.

[B67] Liden R. C. Wayne S. J. Sparrowe R. T. (2000). An examination of the mediating role of psychological empowerment on the relations between the job, interpersonal relationships, and work outcomes. *J. Appl. Psychol.* 85 407–416. 10.1037/0021-9010.85.3.407 10900815

[B68] Lips-Wiersma M. (2002). The influence of “spiritual meaning-making” on career behavior. *J. Manag. Dev.* 21 497–520. 10.1108/02621710210434638

[B66] Lips-Wiersma M. Mcmorland J. (2006). Finding meaning and purpose in boundaryless careers: a framework for study and practice. *J. Humanist. Psychol.* 46 147–167. 10.1177/0022167805283776

[B69] Lips-Wiersma M. Morris L. (2009). Discriminating between ‘meaningful work’ and the ‘management of meaning’. *J. Bus. Ethics* 88 491–511. 10.1007/s10551-009-0118-9

[B71] Lips-Wiersma M. Wright S. (2012). Measuring the meaning of meaningful work: development and validation of the Comprehensive Meaningful Work Scale (CMWS). *Group Organization Manag.* 37 655–685. 10.1177/1059601112461578

[B72] Littman-Ovadia H. Lavy S. (2016). Going the extra mile: perseverance as a key character strength at work. *J. Career Assess.* 24 240–252. 10.1177/1069072715580322

[B73] Littman-Ovadia H. Lavy S. Boiman-Meshita M. (2017). When theory and research collide: examining correlates of signature strengths use at work. *J. Happiness Stud.* 18 527–548. 10.1007/s10902-016-9739-8

[B74] Löwe B. Wahl I. Rose M. Spitzer C. Glaesmer H. Wingenfeld K. (2010). A 4-item measure of depression and anxiety: validation and standardization of the Patient Health Questionnaire-4 (PHQ-4) in the general population. *J. Affect. Disord.* 122 86–95. 10.1016/j.jad.2009.06.019 19616305

[B75] Luhmann N. (1977). Differentiation of society. *Can. J. Sociol.* 2 29–53. 10.2307/3340510

[B76] Lund K. Argentzell E. Leufstadius C. Tjörnstrand C. Eklund M. (2019). Joining, belonging, and re-valuing: a process of meaning-making through group participation in a mental health lifestyle intervention. *Scand. J. Occup. Ther.* 26 55–68. 10.1080/11038128.2017.1409266 29179630

[B77] Lyons-Padilla S. Gelfand M. J. Mirahmadi H. Farooq M. Van Egmond M. (2015). Belonging nowhere: marginalization & radicalization risk among Muslim immigrants. *Behav. Sci. Policy* 1 1–12. 10.1353/bsp.2015.0019

[B78] Lysova E. I. Allan B. A. Dik B. J. Duffy R. D. Steger M. F. (2019). Fostering meaningful work in organizations: a multi-level review and integration. *J. Vocat. Behav.* 110 374–389. 10.1016/j.jvb.2018.07.004

[B79] Maas P. Bühler P. (2015). I-Society: how multi-optionality is pushing individualisation in the digital age. *St. Gallen Bus. Rev.* 2 6–11.

[B80] Mäkikangas A. Hätinen M. Kinnunen U. Pekkonen M. (2011). Longitudinal factorial invariance of the Maslach Burnout Inventory – General Survey among employees with job-related psychological health problems. *Stress Health* 27 347–352. 10.1002/smi.1381

[B81] Martela F. Steger M. F. (2016). The three meanings of meaning in life: distinguishing coherence, purpose, and significance. *J. Posit. Psychol.* 11 531–545. 10.1080/17439760.2015.1137623

[B82] Maslach C. Jackson S. (1981). *The Maslach Burnout Inventory (Human Services Survey).* Palo Alto, CA: Consulting Psychologists Press.

[B83] Maslach C. Jackson S. E. Leiter M. P. (1996). *Maslach Burnout Inventory Manual.* Palo Alto, CA: Consulting Psychologists Press.

[B84] Maslach C. Schaufeli W. B. Leiter M. P. (2001). Job burnout. *Annu. Rev. Psychol.* 52 397–422. 10.1146/annurev.psych.52.1.397 11148311

[B85] May D. R. Gilson R. L. Harter L. M. (2004). The psychological conditions of meaningfulness, safety and availability and the engagement of the human spirit at work. *J. Occup. Organ. Psychol.* 77 11–37. 10.1348/096317904322915892

[B86] McClure J. P. Brown J. M. (2008). Belonging at work. *Hum. Resource Dev. Intl.* 11 3–17. 10.1080/13678860701782261

[B87] Nunnally J. C. (1967). *Psychometric Theory.* New York, NY: McGraw-Hill.

[B88] Nuremberg Institute for Market Decisions and St. Gallen Symposium (2019). *Purpose Beyond Profit: Voices of the Leaders of Tomorrow 2019.* Available online at: www.nim.org/sites/default/files/medien/359/dokumente/-2019_report_lot_web_fin.pdf (accessed July 24, 2020).

[B89] Pavlish C. Hunt R. (2012). An exploratory study about meaningful work in acute care nursing. *Nurs. Forum* 47 113–122. 10.1111/j.1744-6198.2012.00261.x 22512769

[B90] Pedersen H. (2018). “Heidegger on authenticity: the prospect of owning one’s existence,” in *Leadership and the Unmasking of Authenticity*, eds Cusher B. E. Menaldo M. A. (Cheltenham: Edward Elgar Publishing), 57–74. 10.4337/9781786430991.00008

[B91] Podsakoff P. M. MacKenzie S. B. Lee J.-Y. Podsakoff N. P. (2003). Common method biases in behavioral research: a critical review of the literature and recommended remedies. *J. Appl. Psychol.* 88 879–903. 10.1037/0021-9010.88.5.879 14516251

[B92] Pollet E. Schnell T. (2017). Brilliant: but what for? meaning and subjective well-being in the lives of intellectually gifted and academically high-achieving adults. *J. Happiness Stud.* 18 1459–1484. 10.1007/s10902-016-9783-4

[B93] Puchalska-Kamińska M. Czerw A. Roczniewska M. (2019). Work meaning in self and world perspective: a new outlook on the WAMI scale. *Soc. Psychol. Bull.* 14 1–29. 10.32872/spb.v14i1.30207

[B94] Riffkin R. (2014). *In US, 55% of Workers Get Sense of Identity from Their Job.* Available online at: https://news.gallup.com/poll/175400/workers-sense-identity-job.aspx (accessed July 24, 2020).

[B95] Rosso B. D. Dekas K. H. Wrzesniewski A. (2010). On the meaning of work: a theoretical integration and review. *Res. Organ. Behav.* 30 91–127. 10.1016/j.riob.2010.09.001

[B96] Rothausen T. J. Henderson K. E. (2019). Meaning-based job-related well-being: exploring a meaningful work conceptualization of job satisfaction. *J. Bus. Psychol.* 34 357–376. 10.1007/s10869-018-9545-x

[B97] Ruiz-Quintanilla S. A. Claes R. (2000). MOW research programs. *Databases Study Entrep.* 4 335–391. 10.1016/s1074-7540(00)04010-1

[B98] Saarelainen S. M. K. (2018). Lack of belonging as disrupting the formation of meaning and faith: experiences of youth at risk of becoming marginalized. *J. Youth Theol.* 17 127–149. 10.1163/24055093-17021053

[B99] Santana M. C. Fouad N. A. (2017). Development and validation of a self-care behavior inventory. *Train. Educ. Prof. Psychol.* 11 140–145. 10.1037/tep0000142

[B100] Savvides E. Stavrou E. (2020). “Purpose, meaning, joy, and fulfilment at work,” in *The Palgrave Handbook of Workplace Well-Being*, ed. Dhiman S. (London: Palgrave Macmillan), 1–27. 10.1007/978-3-030-02470-3_36-1

[B101] Scannell E. D. Allen F. C. L. Burton J. (2002). Meaning in life and positive and negative well-being. *N. Am. J. Psychol.* 4 93–112.

[B102] Schaufeli W. B. Leiter M. P. Maslach C. Jackson S. E. (1996). “Maslach Burnout Inventory – General Survey,” in *The Maslach Burnout Inventory: Test Manual*, eds Maslach C. Jackson S. E. Leiter M. P. (Palo Alto, CA: Consulting Psychologists Press), 103–111. 10.1016/j.burn.2014.09.001

[B103] Schnell T. (2004/2009). *Implizite Religiosität – Zur Psychologie des Lebenssinns.* Lengerich: Pabst Science Publishers.

[B104] Schnell T. (2009). The Sources of Meaning and Meaning in Life questionnaire (SoMe): relations to demographics and well-being. *J. Posit. Psychol.* 4 483–499. 10.1080/17439760903271074

[B105] Schnell T. (2011). Individual differences in meaning-making: considering the variety of sources of meaning, their density and diversity. *Pers. Individ. Differ.* 51 667–673. 10.1016/j.paid.2011.06.006

[B106] Schnell T. (2014). “An empirical approach to existential psychology: meaning in life operationalized,” in *Conceptions of Meaning*, eds Kreitler S. Urbanek T. (New York, NY: Nova Science), 173–194.

[B107] Schnell T. (2018). “Von Lebenssinn und Sinn in der Arbeit. Warum es sich bei beruflicher Sinnerfüllung nicht um ein nettes Extra handelt,” in *Fehlzeiten-Report 2018*, eds Badura B. Ducki A. Schröder H. Klose J. Meyer M. (Berlin: Springer Verlag), 11–21. 10.1007/978-3-662-57388-4_2

[B108] Schnell T. (2020). *The Psychology of Meaning in Life.* Abingdon: Routledge.

[B109] Schnell T. Becker P. (2007). *Der Fragebogen zu Lebensbedeutungen und Lebenssinn (LeBe).* Göttingen: Hogrefe.

[B110] Schnell T. Höge T. Pollet E. (2013). Predicting meaning in work: theory, data, implications. *The J. Posit. Psychol.* 8 543–554. 10.1080/17439760.2013.830763

[B111] Schnell T. Höge T. Weber W. G. (2019). “Belonging’ and its relationship to the experience of meaningful work,” in *The Oxford Handbook of Meaningful Work*, eds Yeoman R. Bailey K. Madden A. Thompson M. (Oxford: Oxford University Press), 165–185.

[B112] Seeman M. (1959). On the meaning of alienation. *Am. Sociol. Rev.* 24 783–791. 10.2307/2088565

[B113] Steger M. F. Dik B. J. (2009). If one is looking for meaning in life, does it help to find meaning in work? *Appl. Psychol. Health Well-Being* 1 303–320. 10.1111/j.1758-0854.2009.01018.x

[B114] Steger M. F. Dik B. J. Duffy R. D. (2012). Measuring meaningful work: the work and meaning inventory (WAMI). *J. Career Assess.* 20 322–337. 10.1177/1069072711436160

[B115] Stillman T. F. Baumeister R. F. (2009). Uncertainty, belongingness, and four needs for meaning. *Psychol. Inquiry* 20 249–251. 10.1080/10478400903333544

[B116] Supanti D. Butcher K. (2019). Is corporate social responsibility (CSR) participation the pathway to foster meaningful work and helping behavior for millennials? *Intl. J. Hosp. Manag.* 77 8–18. 10.1016/j.ijhm.2018.06.001

[B117] Tabachnick B. G. Fidell L. S. Ullman J. B. (2007). *Using Multivariate Statistics.* Boston, MA: Pearson.

[B118] The Editors of Encyclopaedia Britannica (2018). *Work*. *in Encyclopædia Britannica.* Available online at: https://www.britannica.com/topic/work-economics (accessed November 1, 2020).

[B119] Tims M. Derks D. Bakker A. B. (2016). Job crafting and its relationships with person–job fit and meaningfulness: a three-wave study. *J. Vocat. Behav.* 92 44–53. 10.1016/j.jvb.2015.11.007

[B120] Unterrainer C. Jeppesen H. J. Jønsson T. (2013). Different forms of job satisfaction: does job satisfaction mean satisfied employees? *Psyke Logos* 34 398–419.

[B121] Verdorfer A. P. Steinheider B. Burkus D. (2015). Exploring the socio-moral climate in organizations: an empirical examination of determinants, consequences, and mediating mechanisms. *J. Bus. Ethics* 132 233–248. 10.1007/s10551-014-2319-0

[B122] Wrzesniewski A. McCauley C. Rozin P. Schwartz B. (1997). Jobs, careers, and callings: People’s relations to their work. *J. Res. Pers.* 31 21–33. 10.1006/jrpe.1997.2162

[B123] XING (2019). *Xing Gehaltsstudie 2019.* Available online at: https://corporate.xing.com/fileadmin/user_upload/XING-Gehaltsstudie-2019-DE.pdf (accessed July 24, 2020).

[B124] Yang K. Girgus J. S. (2019). Are women more likely than men are to care excessively about maintaining positive social relationships? a meta-analytic review of the gender difference in sociotropy. *Sex Roles* 81 157–172. 10.1007/s11199-018-0980-y

[B125] Yeoman R. Bailey C. Madden A. Thompson M. (eds) (2019). *The Oxford Handbook of Meaningful Work.* Oxford: Oxford University Press.

