# Supplementary material for: ME-Work: Development and Validation of a Modular Meaning in Work Inventory
Source: Front Psychol. 2020 Dec 7;11:599913. doi: 10.3389/fpsyg.2020.599913 (PMC7793865; doi:10.3389/fpsyg.2020.599913)
Supplement: Supplementary file 1 [file Table_1.DOCX]

**APPENDIX A**

**ME-Work, German version**

Lesen Sie folgende Aussagen bitte genau durch und kreuzen Sie an, inwiefern Sie diesen zustimmen.

Antwortformat: 0 (stimme überhaupt nicht zu) bis 5 (stimme vollkommen zu)

**Kohärenz**

1. Meine Arbeitstätigkeit entspricht meinen Interessen.
2. Die Rolle, die ich in meiner Arbeit einnehme, passt gut zu meinen persönlichen Eigenschaften.
3. Meine beruflichen Tätigkeiten passen gut zu dem, was ich mir im Leben vorgenommen habe.

**Bedeutsamkeit**

1. Meine Arbeit macht die Welt ein kleines bisschen besser.
2. Durch meine berufliche Tätigkeit leiste ich einen wertvollen Beitrag für die Gesellschaft.
3. Meine Arbeit bereichert das Leben von anderen.

**Orientierung**

1. An meinem Arbeitsplatz geht Profit vor Menschlichkeit. (-)
2. Mein Arbeitgeber tut etwas dafür, soziale Probleme zu lösen.
3. Meinem Arbeitgeber ist es wichtiger, dass Aufgaben sorgfältig erledigt werden als in möglichst kurzer Zeit.
4. Mein Arbeitgeber hat das Wohl der Gesellschaft im Sinn.

**Zugehörigkeit**

1. Ich bin gut eingebunden in die kollegiale Gemeinschaft.
2. In Gesellschaft meiner KollegInnen fühle ich mich wohl.
3. Bei der Arbeit sind wir ein super Team.

**Sinnvolle Arbeit**

1. Die Tätigkeiten, die ich an meiner Arbeit ausführe, erscheinen mir sinnvoll.
2. Ich sehe einen Sinn in meiner Arbeit.
3. Mein Beruf erscheint mir sinnvoll.

**Sinnleere Arbeit**

1. Ich stecke beruflich in einer Sinnkrise.
2. Wenn ich über den Sinn meiner Arbeit nachdenke, empfinde ich nur Leere.
3. Ich leide darunter, dass ich in meiner Arbeit keinen Sinn entdecken kann.

**Beruf als Sinnquelle**

1. Ich kann mich in meinem Beruf selbst verwirklichen.
2. Mein Beruf erfüllt mich.
3. Ich kann an meiner Arbeit wachsen.
4. Meine Arbeitstätigkeit verleiht meinem Leben Sinn.

**APPENDIX B**

**ME-Work, English translation**

Please read the following statements carefully and indicate to what extent you agree with them.

Response format: 0 (don’t agree at all) to 5 (agree completely)

**Coherence**

1. My work reflects my interests.
2. The position I hold in my job fits well with my personal characteristics.
3. My professional activities fit well with what I have set out to do in life.

**Significance**

1. My work makes the world a little bit better.
2. Through my professional activity I make a valuable contribution to society.
3. My work enriches the lives of others.

**Purpose**

1. In my workplace, profit comes before humanity. (-)
2. My employer is involved in solving social problems.
3. It is more important to my employer that tasks are carried out carefully than in the shortest possible time.
4. My employer cares about the welfare of society.

**Belonging**

1. I am well integrated into the collegial community.
2. I feel comfortable in the company of my colleagues.
3. We are a great team at work.

**Meaningful work**

1. The work I am doing on my job makes sense to me.
2. I see meaning in my work.
3. I find my profession meaningful.

**Meaningless work**

1. I'm in a career crisis.
2. When I think about the meaning of my work, I feel nothing but emptiness.
3. I feel pain from finding no meaning in my work.

**Work as a source of meaning**

1. I can achieve self-realisation in my work.
2. My work fulfils me.
3. I can grow through my work.
4. My professional activity gives meaning to my life.
